# Coil Embolization and Open Splenectomy for a Massive Splenic Vein Aneurysm: A Case Report

**DOI:** 10.1155/crvm/6210301

**Published:** 2026-10-17

**Authors:** Kaitlyn Vu, Logan Brown, Blake Durrence, Tilak Patel, Robert Larsen, Stephanie Gordy

**Affiliations:** ^1^ Mercer University School of Medicine Savannah Georgia USA mercer.edu; ^2^ Department of Surgery Memorial Health University Medical Center Savannah Georgia USA

**Keywords:** case report, coil embolization, portal hypertension, splenectomy, splenic vein aneurysm

## Abstract

**Background:**

Splenic vein aneurysms (SVAs) are a rare vascular anomaly of the portal system that are often asymptomatic and found incidentally on imaging. Intervention is reserved for aneurysms that are enlarging, thrombosed, symptomatic, or ruptured to avoid catastrophic complications. This can range from endovascular to open, complex procedures. Here, we present the case of a massive, symptomatic, partially thrombosed SVA that required a hybrid approach involving both minimally invasive and open surgery.

**Case Presentation:**

A 56‐year‐old woman presented to the emergency department for an infected right foot wound and was admitted for intravenous antibiotics and surgical debridement. While hospitalized, she had a worsening transaminitis with associated abdominal pain, prompting investigation with a computed tomography (CT) scan, which identified a massively dilated, partially thrombosed SVA measuring 18 cm. Given the enormous size of the aneurysm and substantial risk of impending rupture, urgent surgical intervention was planned. Preoperative coil embolization of the splenic artery was performed first to reduce the arterial inflow to the SVA and to minimize the risk of intraoperative hemorrhage during open resection. Three days after embolization, the patient underwent open splenectomy with aneurysmectomy for definitive management. The patient′s postoperative course was complicated by a bleeding gastric ulcer that was successfully cauterized, and she was discharged home on postoperative Day 10 after receiving the recommended postsplenectomy vaccines.

**Conclusion:**

No standard treatment algorithm exists for SVAs, as management is individualized, and treatment evidence is limited to case reports and series. This patient had one of the largest SVAs ever recorded, and management was based on the acuity of symptoms, size, and rupture risk of the aneurysm sac. This case also demonstrated the success of a novel management approach to massive SVAs, using preoperative embolization of the splenic artery followed by open resection without portal reconstruction.

## 1. Introduction

An aneurysm is an abnormal bulging or uniform dilation of a blood vessel due to an intrinsic weakness in the vessel wall [1]. Although aneurysms can occur in both arteries and veins, venous aneurysms are rare because of the low‐pressure system and low wall stress. Arterial mesenteric aneurysms occur from trauma, infection, atherosclerosis, or connective tissue disorders. Mesenteric vein aneurysms are typically located in the portal system in the setting of portal hypertension or prior pancreatitis [2]. They can be congenital or acquired and are often asymptomatic. Complications such as thrombosis, compression of adjacent structures, and rupture can occur. Although management guidelines exist for the management of visceral arterial aneurysms, there is no standard treatment algorithm regarding the management of venous aneurysms. Management ranges from nonoperative management to aneurysm excision depending on the size and location of the vascular abnormality [2, 3].

Here, we present the case of a 56‐year‐old woman who was diagnosed with an enormous splenic vein aneurysm (SVA) measuring 18 cm on a computed tomography (CT) scan, which was discovered during a hospitalization for a lower extremity wound infection. Given the substantial risk of impending rupture, the decision was made to surgically resect the aneurysm while the patient remained hospitalized. A hybrid approach was used involving preoperative coil embolization of the splenic artery followed by open splenectomy with aneurysmectomy. By embolizing the splenic artery first, arterial inflow and expected venous outflow to the SVA would be reduced, stabilizing the aneurysm and allowing for better hemorrhage control intraoperatively during the open resection. Although this combined approach is often employed in the management of arterial visceral aneurysms, there have been few documented reports of this approach in the management of SVAs.

## 2. Case Presentation

The patient was a 56‐year‐old woman with a history of tobacco use and a prior cholecystectomy complicated by a retained gallstone requiring endoscopic retrograde cholangiopancreatography (ERCP), who presented to the emergency department with an infected right foot wound and was admitted for intravenous antibiotic therapy and surgical debridement. Between hospital Days 3 and 5, she began to endorse vague abdominal pain with an associated transaminitis, and an abdominal ultrasonography (US) was performed for further evaluation. This imaging showed a thick‐walled cystic structure with internal debris and peripheral vascularity in the left upper quadrant. A follow‐up CT scan revealed a massive, partially thrombosed SVA measuring 18 × 13 cm (Figure 1). A portal system duplex was also obtained, which showed no evidence of portal hypertension. Given the unprecedented size of the aneurysm and the high risk of impending rupture, surgical intervention was planned while the patient remained acutely hospitalized.

**Figure 1:**
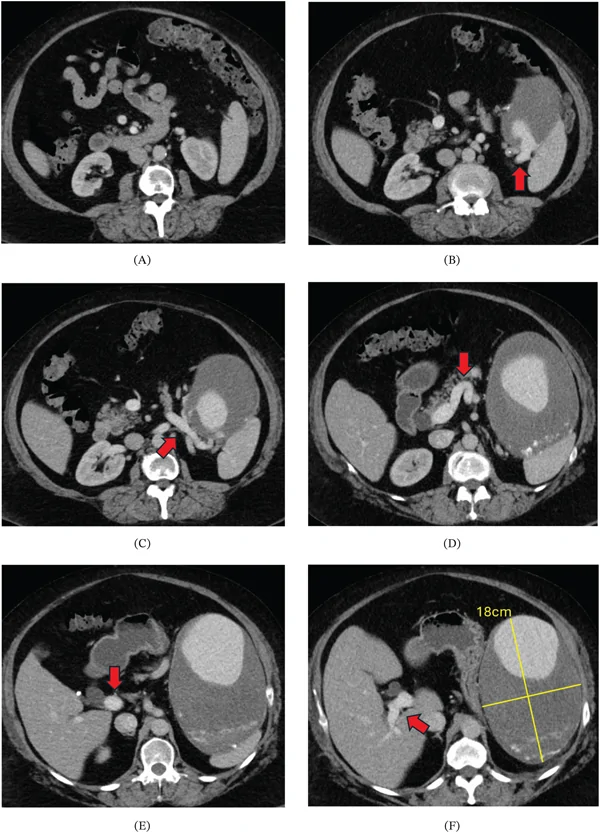
Images of the patient′s first computed tomography results. (A) Axial view of the patient′s normal appearing spleen. (B) Initial view of the partially thrombosed splenic vein aneurysm. (C–E) Tortuous path of the splenic vein, indicated by the red arrow. (F) Confluence of the splenic vein with the portal system and massively dilated venous aneurysm, 18 cm in max diameter on preoperative imaging.

Interventional radiology (IR) was consulted to determine if the patient would be a candidate for preoperative coil embolization of the splenic artery. After obtaining informed consent, the patient was taken to the angiography suite, and her right common femoral artery (CFA) was accessed under direct US visualization. A 5‐French (F) catheter was advanced into the splenic artery, and an angiogram was performed, confirming the CT findings. Coil embolization of the splenic artery was then performed, with a total of eight detachable coils ranging in size from 8 to 13 mm. A second angiogram showed a significant decrease in arterial in‐line flow to the spleen (Figure 2). The arteriotomy was closed with an Angio‐Seal closure device, and there were no immediate complications. Two days after the splenic artery coil embolization, the patient received the recommended splenic vaccinations, and on hospital Day 10, she was taken to the operating room for an open splenectomy and aneurysmectomy.

**Figure 2:**
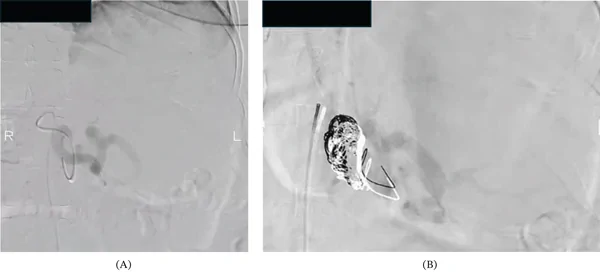
(A) Angiogram of the splenic artery with contrast filling the superior edge of the massive SVA. (B) Decrease in contrast medium flow into the SVA following coil embolization of the splenic artery.

The abdomen was entered via a left subcostal incision, and after inspection, the splenic flexure was mobilized by incising the avascular plane along the white line of Toldt. The gastrocolic ligament was transected, and the lesser sac was opened. Using a bipolar vessel sealer device, the short gastric arteries were ligated. The aneurysm was then circumferentially freed, taking care not to enter the sac. The proximal splenic vein and artery were identified and divided with an endoscopic stapler. The spleen was then fully mobilized by taking down the remaining ligaments (Figure 3). A liver biopsy was also performed because of the nodular appearance of the liver. Hemostasis was achieved and the abdomen was closed in layers.

**Figure 3:**
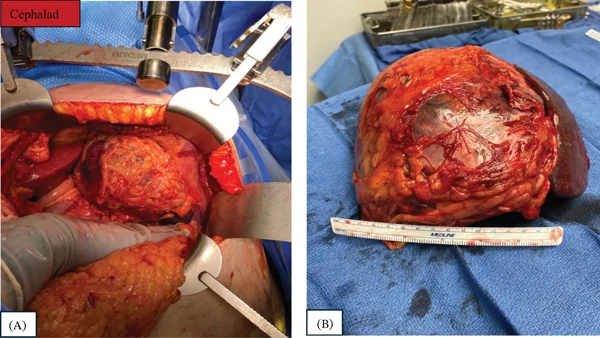
(A) Intraoperative view of the aneurysm during the circumferential dissection. (B) En bloc removal of the spleen and aneurysm, measuring 19.5 × 17.5 × 10.5 cm on gross pathology.

Between postoperative Days 6 and 8, the patient experienced episodes of melena requiring transfusion of blood products. Gastroenterology (GI) was consulted, and an esophagogastroduodenoscopy (EGD) was performed. A bleeding gastric ulcer was identified and successfully cauterized with a gold probe. The patient was discharged home on postoperative Day 10, with final pathology revealing a histologically unremarkable spleen with a large, thrombosed SVA and a benign bile duct hamartoma. She was seen on postoperative Day 21 for a routine postoperative visit and was recovering well overall. She was tolerating a diet with no clinical signs of bleeding, and no further imaging was pursued. A complete and detailed outline of the patient′s hospital course is listed in Figure 4.

**Figure 4:**
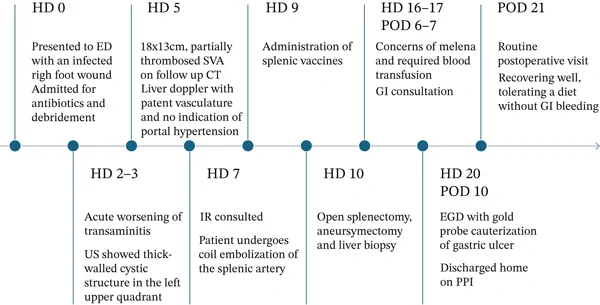
Detailed outline of the relevant clinical events that transpired during the patient′s acute hospitalization.

## 3. Discussion

SVAs are a rare example of extrahepatic portal venous aneurysms. These aneurysms are usually diagnosed in the setting of portal hypertension, liver disease, or pancreatitis [3, 4]. SVAs are often asymptomatic and discovered incidentally on imaging. They may also cause vague abdominal pain from thrombosis, obstructive symptoms from organ compression, or life‐threatening hemorrhage due to rupture [2, 5–7]. CT scans with contrast medium are beneficial to delineate the relevant anatomy. Doppler US can be used to assess vein patency, blood flow velocity and direction, and cystic characteristics of the aneurysm [3]. When noninvasive modalities remain inconclusive, angiography is the gold standard as it offers both diagnostic and therapeutic techniques [8].

Management of SVAs is guided by the patient′s hemodynamic stability, aneurysm characteristics, and portal hemodynamics. In hemodynamically stable patients without portal hypertension, SVAs can be managed conservatively with serial imaging. Intervention is reserved for symptomatic aneurysms with rapid interval growth, thrombosis, mass effect, or concern for rupture [2–4]. Endovascular options such as embolization and stenting are preferred in anatomically suitable patients because they preserve splenic function and are associated with lower perioperative morbidity and quicker recovery times [8, 9]. Open surgery including aneurysmectomy with or without venous reconstruction, combined with splenectomy and occasionally distal pancreatectomy, is typically reserved for impending aneurysm rupture, complex anatomy, hemorrhage, or failed/contraindicated endovascular therapy [2–4].

The patient denied any history of trauma but did have a history of biliary pathology, which could have led to the initial development of the SVA. She had previously undergone a laparoscopic cholecystectomy for cholecystitis, but her postoperative course was complicated by a retained stone in her biliary system that required a postoperative ERCP. Although she was never officially diagnosed, it is possible that she developed pancreatitis, either from the retained gallstone or postoperatively following the ERCP, leading to the formation of the SVA. It was then subsequently discovered during her acute hospitalization for sepsis, after it thrombosed and she became symptomatic with abdominal pain and worsening transaminitis, leading to a more in‐depth work‐up.

Although the patient was hemodynamically stable without portal hypertension, we did not believe that conservative management with serial imaging was appropriate after discovering the gigantic aneurysm. At 18 cm on imaging, it was one of the largest documented SVAs ever recorded [2, 4, 5, 10]. Given its sheer size, high risk for imminent rupture, partial thrombosis, and symptomatic abdominal pain, the best course of action was to pursue surgical intervention while the patient remained hospitalized.

A hybrid approach using both endovascular and open surgical techniques was used for several reasons. First, we did not believe that endovascular intervention alone would be sufficient to isolate the SVA and it would not significantly reduce her rupture risk. Aneurysmectomy with splenectomy would be the only way to definitively reduce that risk [2, 4, 5]. However, by performing preoperative coil embolization of the splenic artery, the arterial inflow to the spleen would be significantly reduced. Although this would not address any venous backfilling, it would stabilize the size of the aneurysm and allow for proximal arterial control during the open resection, helping to minimize intraoperative hemorrhage. This strategy is heavily favored in the management of splenic artery aneurysms, but is not well described in the management of SVAs given there are no dedicated treatment algorithms [8, 9, 11].

Finally, an open resection of both the aneurysm and spleen provided the best visualization of this patient′s complex anatomy. There was no involvement of the pancreas, and a clean dissection plane was able to be created to avoid performing a distal pancreatectomy. We opted to forgo venous reconstruction following the resection as there appeared to be adequate venous drainage with no signs of portal hypertension.

## 4. Conclusion

SVAs are a rare vascular anomaly that usually occur in the setting of portal hypertension. They can be diagnosed with a wide variety of imaging modalities, and the overall prognosis is favorable. There is no standard algorithm regarding the management of SVAs, with treatment options including conservative management, endovascular procedures and surgical section depending on a multitude of factors. This patient developed a gigantic, partially thrombosed, and symptomatic SVA, which prompted swift and aggressive intervention with preoperative endovascular coil embolization followed by open splenectomy and aneurysmectomy. This case highlights the novel combined approach in one of the largest reported SVAs and adds to the growing literature regarding the management of massive SVAs.

**Nomenclature**
CT: computed tomography
ED: emergency department
EGD: esophagogastroduodenoscopy
GI: gastroenterology
HD: hospital day
IR: interventional radiology
POD: postoperative day
PPI: proton pump inhibitor
SVA: splenic vein aneurysm
US: ultrasonography

## Funding

No funding was received for this manuscript.

## Disclosure

The views expressed in this publication represent those of the author(s) and do not necessarily represent the official views of HCA Healthcare or any of its affiliated entities.

## Ethics Statement

Institutional Review Board (IRB) approval was waived for this single case report. Informed written consent was obtained directly from the patient for publication regarding the case details and included images. The patient′s identity was always protected throughout the entire process.

## Conflicts of Interest

The authors declare no conflicts of interest.

## Supporting information


**Supporting Information** Additional supporting information can be found online in the Supporting Information section. CARE checklist.

## Data Availability

Given this is a care report, we have no data to support.
