# Supplementary material for: Coil Embolization and Open Splenectomy for a Massive Splenic Vein Aneurysm: A Case Report
Source: Case Rep Vasc Med. 2026 Oct 17;2026:6210301. doi: 10.1155/crvm/6210301 (PMC13676035; doi:10.1155/crvm/6210301)
Supplement: Supplementary file 1 [file CRVM-2026-6210301-s001.docx]

103-111

106-111

NA

NA

Na

162-171

132-140

112-161

112-161

103-111

Na

83-102

83-102

78-79

75-80

5-80

Figure 4

75-80

72-73

71-73

72-77

71-73

59-69

41-46

33-40

29-33

27-28

22

1-2

NA

✓
